# High-sensitivity MALDI-TOF MS quantification of anthrax lethal toxin for diagnostics and evaluation of medical countermeasures

**DOI:** 10.1007/s00216-015-8509-5

**Published:** 2015-02-12

**Authors:** Anne E. Boyer, Maribel Gallegos-Candela, Conrad P. Quinn, Adrian R. Woolfitt, Judith O. Brumlow, Katherine Isbell, Alex R. Hoffmaster, Renato C. Lins, John R. Barr

**Affiliations:** 1 Centers for Disease Control and Prevention, Atlanta, GA 30341 USA; 2 Battelle-Technical On-Site Professional Services, Atlanta, GA 30329 USA

**Keywords:** Anthrax, MALDI-TOF MS, Quantification, Lethal toxin, Lethal factor, Protective antigen, Diagnostic, *Bacillus anthracis*

## Abstract

Inhalation anthrax has a rapid progression and high fatality rate. Pathology and death from inhalation of *Bacillus anthracis* spores are attributed to the actions of secreted protein toxins. Protective antigen (PA) binds and imports the catalytic component lethal factor (LF), a zinc endoprotease, and edema factor (EF), an adenylyl cyclase, into susceptible cells. PA-LF is termed lethal toxin (LTx) and PA-EF, edema toxin. As the universal transporter for both toxins, PA is an important target for vaccination and immunotherapeutic intervention. However, its quantification has been limited to methods of relatively low analytic sensitivity. Quantification of LTx may be more clinically relevant than LF or PA alone because LTx is the toxic form that acts on cells. A method was developed for LTx-specific quantification in plasma using anti-PA IgG magnetic immunoprecipitation of PA and quantification of LF activity that co-purified with PA. The method was fast (<4 h total time to detection), sensitive at 0.033 ng/mL LTx in plasma for the fast analysis (0.0075 ng/mL LTx in plasma for an 18 h reaction), precise (6.3–9.9 % coefficient of variation), and accurate (0.1–12.7 %error; *n* ≥ 25). Diagnostic sensitivity was 100 % (*n* = 27 animal/clinical cases). Diagnostic specificity was 100 % (*n* = 141). LTx was detected post-antibiotic treatment in 6/6 treated rhesus macaques and 3/3 clinical cases of inhalation anthrax and as long as 8 days post-treatment. Over the course of infection in two rhesus macaques, LTx was first detected at 0.101 and 0.237 ng/mL at 36 h post-exposure and increased to 1147 and 12,107 ng/mL in late-stage anthrax. This demonstrated the importance of LTx as a diagnostic and therapeutic target. This method provides a sensitive, accurate tool for anthrax toxin detection and evaluation of PA-directed therapeutics.

**Graphical Abstract:**
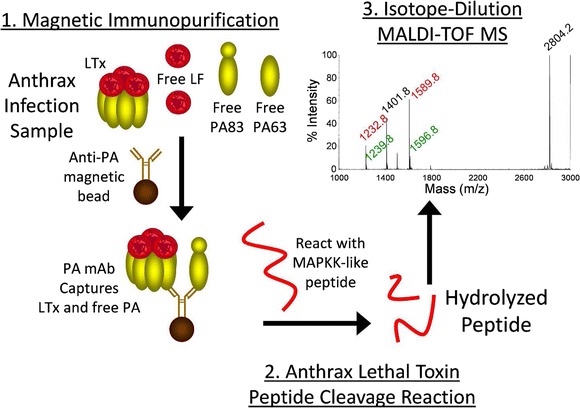
Method schematic for analysis of anthrax lethal toxin activity by ID-MALDI-TOF MS

Method schematic for analysis of anthrax lethal toxin activity by ID-MALDI-TOF MS

## Introduction

*Bacillus anthracis* produces two binary toxins associated with the pathogenesis of anthrax [1]. Protective antigen (PA) is an 83-kDa protein responsible for cell binding and target cell translocation of catalytic toxin components lethal factor (LF) and edema factor (EF). Thus, PA is the universal target for development of anti-toxin treatments and vaccines since blocking it prevents cellular intoxication. Methods described for detecting PA include ELISA, europium nanoparticle-based immunoassay, time-resolved fluorescence immunoassay [2–5], electro-chemiluminescence, metal-enhanced fluorescence, AlphaLISA, and surface plasmon resonance [6–9]. However, these methods can be lacking in precision, sensitivity, and quantitative accuracy, and in some, their utility has not been verified by matrix testing or application to infection samples.

Detection of PA during infection can be complex as it may exist in many forms. During cellular intoxication, PA83 binds to cell surface receptors CMG2 and TEM8 [10, 11], where it is cleaved by furin-like proteases, releasing its 20-kDa amino terminus, leaving 63 kDa (PA63) bound to the cell surface [1]. PA63 forms heptameric and octameric complexes [12, 13], which are capable of binding up to four molecules of the catalytic toxin components, edema factor (EF), and lethal factor (LF) [13, 14]. EF bound to PA is described as edema toxin and LF bound to PA as lethal toxin (LTx). The PA63-EF/LF complexes are internalized by clathrin-mediated endocytosis [15]. The low pH within the endosome triggers conformational changes in the PA63 oligomer, leading to pore-formation and translocation of EF/LF into the cell cytoplasm [1]. Within the cell, EF, an adenylate cyclase and LF, a zinc-dependent endoproteinase cause irreversible changes in their known substrates, adenosine tri-phosphate and mitogen-activated protein kinase (MAPKK), respectively [16, 17].

Previous studies found that PA83 is activated to PA63 by proteases in the blood [18, 19]. Serum protease-activated PA63 was shown to bind LF and form fully functional LTx [20]. LTx was also identified in terminal blood of infected rabbits and guinea pigs [20]. None of the PA was found as PA83 [18, 20]. These findings suggest LTx is a potential diagnostic biomarker and distinct therapeutic target. However, LTx has not been detected or measured prior to the moribund and terminal stages. Thus, until this work, it was not known whether LTx is present in early infection.

We previously described an isotope-dilution (ID) matrix-assisted laser-desorption ionization time-of-flight (MALDI-TOF) mass spectrometry method that quantifies total LF (free LF+LTx) [21]. The method incorporates three steps, antibody capture, LF peptide substrate cleavage, and MS detection of cleaved peptide. These steps each provide a level of specificity and sensitivity and matrix detection limits of 0.005 pg/mL [21–24].

The utility of total LF measurement was demonstrated in two circumstances. The first was characterization of triphasic toxemia during the course of experimental inhalation anthrax [25]. The second was characterization of toxin clearance during treatment of naturally occurring inhalation anthrax which showed that total LF declined gradually with antibiotic treatment and rapidly with anthrax immune globulin intravenous (AIGIV) anti-toxin treatment [26, 27]. AIGIV is composed of immune plasma from individuals immunized with the anthrax vaccine adsorbed (BioThrax®) [28]. The therapeutic component is predominantly anti-PA IgG which binds PA and associated proteins such as LF and EF, targeting them for removal from circulation through Fc-mediated immune mechanisms. Monitoring LF provides an indirect measurement of toxin clearance since it is not known how much LF is PA associated. An LTx-specific method would provide a direct measure of PA-specific toxin clearance. Importantly, it could also determine the presence of LTx throughout infection and its potential as a therapeutic and diagnostic target.

**Fig. 1:**
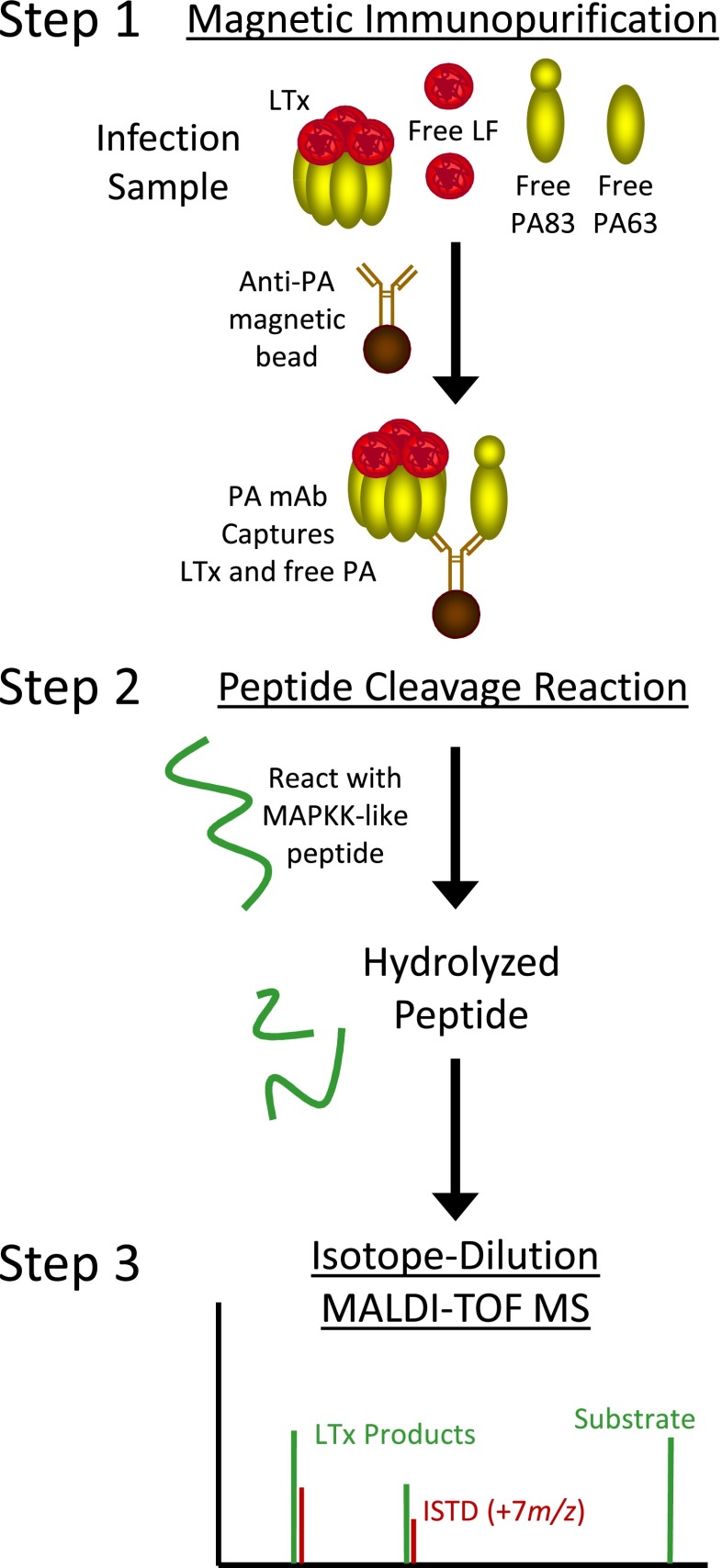
Schematic of the three-step LTx method. LTx-specific activity is analyzed using anti-PA mAb's on magnetic beads to capture LTx complex (*step 1*) followed by incubation of LTx on the bead with a MAPKK-like peptide substrate which it hydrolyzes into two distinct smaller peptide products (*step 2*). The products are detected and quantified by isotope-dilution MALDI-TOF MS for a measure of the LTx in a sample (*step 3*)

Here, we describe a three-step method to detect and measure LTx. Anti-PA mAb magnetic beads were used to immunoprecipitate PA (Fig. 1, step 1). Immobilized LTx is reacted with and hydrolyzes an optimized peptide substrate (step 2). Cleaved peptide products are detected and quantified by MALDI-TOF MS (step 3). This provided measurement of LF that co-purified with activated PA63, quantifying LTx complex. This study describes development, validation, and application of the LTx method. The method was sensitive, specific, and precise and demonstrated for the first time that PA63 as LTx is present in early anthrax.

## Materials and methods

### Reagents

Materials and reagents were obtained from Sigma-Aldrich (St Charles, MO) except where indicated. LF, EF, PA83, and PA63 were obtained from List Biological Laboratories (Campbell, CA). Normal North American (NNA) human ten-donor-pooled plasma and serum and plasma from 100 individual NNA donors were obtained from Interstate Blood Bank (Memphis, TN). AIGIV was obtained from the Division of Strategic National Stockpile, CDC (Atlanta, GA).

### Animal care and study protocol

The inhalation anthrax study in 24 rhesus macaques (*Macaca mulatta*) was conducted at the Battelle Biomedical Research Center (Columbus, OH) as previously described and approved by both Battelle and the Centers for Disease Control and Prevention Institutional Animal Care and Use Committees [25]. This two-part study was approved in 2006 (CDC IACUC protocol no. 1459BOYMONX and Battelle MREF protocol no. 570) and carried out in 2006–2007. Sufficient sample volumes generated from this approved protocol were remaining and used for this study. Animals were challenged with 275 ± 72 LD_50_ aerosolized *B. anthracis* Ames spores by inhalation (target dose 200 LD_50_). Whole blood and plasma were collected in EDTA tubes at −30 days and 12, 18, 24, 36, 48, 72, 96, and 120 h, and serum was collected for five animals as described previously [25]. Plasma/serum was filter sterilized and culture confirmed before shipment. Culture and PCR of the protective antigen gene (*pagA*) were described previously [25]. Total LF and LTx analysis was determined in two animals, 16N and 89N, over the course of infection. An additional 22 post-spore exposure samples were analyzed. Pre- and post-antibiotic treatment samples were available for six animals treated with Ciprofloxacin at 20 mg/kg twice daily for 14 days in surviving animals. Treatment commenced at 48 h post-challenge in four animals and at 72 h in two. Four of six survived.

### Magnetic antibody beads

Anti-PA mouse monoclonal antibodies (mAb) AVR1046 and two anti-LF mAb (AVR1674 and AVR1675) were linked to Tosyl-activated magnetic beads (MB; Life Technologies, Grand Island, NY) according to the manufacturer’s instructions yielding anti-PA and dual anti-LF MBs. Kingfisher 96 magnetic particle processor (Thermo-Fisher Scientific, Waltham, MA) was used for all MB handling.

### Antibody specificity

Specificity of anti-PA and anti-LF MBs was demonstrated for LTx, LF, and PA. PA63/LF were mixed at a *w*/*w* ratio of 10:1 to form LTx complex. PA83 and LF were mixed as a non-complexed control. Mixtures were incubated at room temperature (RT) for 30 min. LF, PA63, PA83, LF/PA63, and LF/PA83 at 0.5 μg LF and 5 μg PA83 or PA63 were immunoprecipitated with anti-LF and anti-PA MBs, eluted with 50 % acetonitrile, 2 mM HCl for 1 min, dried by centrifugal evaporation, reconstituted in sample buffer, analyzed by 5–14 % SDS-polyacrylamide gel electrophoresis (PAGE; Life Technologies), and visualized by silver stain (SS; Protea Biosciences, Morgantown, WV).

### Lethal toxin standard preparation and characterization

LTx was formed by mixing PA63 and LF at *w*/*w* ratios of 0:1 to 60:1 PA63/LF, incubating at RT for 30 min. Mixtures were analyzed for free LF levels by non-denaturing PAGE (Life Tech) and SS. A ratio of 50:1 adsorbed the maximum amount of LF. The final LTx standard material was similarly prepared with 25 μg PA63/0.5 μg LF for 500 ng/μL PA63 and 10 ng/μL LF as LTx, then diluted as described below to make standard and quality control materials.

The LTx standard material was analyzed by non-denaturing PAGE to quantify the “free” LF and correct standards. Gel lanes contained 7.5 μg PA63, 25, 12.5, 6.25, and 3.125 ng LF and the LTx standard material with 150 ng LF and 7.5 μg PA63 to visualize and quantify the small amount of unbound LF. The gel was scanned and bands intensities quantified (Softmax Pro, Molecular Devices, Sunnydale, CA). Free LF intensities were fitted to a linear regression (*r* ^2^ = 0.996), and free LF in the LTx lane was calculated from the linear regression.

The qualitative efficiency of anti-PA purification of the LTx standard material in plasma in the presence of the excess PA63 was determined by comparison with anti-LF purification. The LTx standard material at 100, 50, 25, and 12.5 ng LF and 50 times PA63 each in plasma sample were immunoprecipitated by anti-PA and anti-LF MBs and eluted and analyzed by SDS-PAGE/SS.

Standard and quality control (QC) materials in plasma were prepared from the characterized material with free LF corrected values of 24.3 to 0.0025 ng/mL and three QCs with 5.82 ng/mL (QCH), 0.582 ng/mL (QCM), and 0.0582 ng/mL (QCL) LTx.

### LTx quantification

For LTx analysis, 50 μL of sample was diluted 1:10 in 450 μL phosphate-buffered saline with 0.05 % Tween20 (PBST), mixed with 6.25 μL anti-PA-MBs for 1 h, washed two times 2 min each in 1.0 mL PBST, then in 200 and 100 μL dH_2_O for 1 min each, resuspended in 30 μL reaction buffer with the peptide substrate and reacted for 2 and 18 h at 37 °C. After the 2-h reaction, 3 μL of sample was mixed with α-cyano-4-hydroxycinnamic acid (CHCA) at 5 mg/mL in 50 % acetonitrile, 0.1 % trifluoroacetic acid, and 1 mM ammonium phosphate (CHCA-MALDI matrix) and isotopic internal standard peptides (ISTD) and analyzed by ID-MALDI-TOF/MS as described previously [22, 23]. The remaining reaction was incubated for analysis at 18 h. The LTx cleaved peptide MS peak area/ISTD peak area (area ratio) was plotted versus LTx standard concentration with dual log_10_ transformation and 5PL robust quantification.

### LTx method validation

Recovery for this method compared activities for 0.5 ng LF and LTx (0.5 ng LF + 1 ng PA63) both without capture and with anti-PA purification. Precision, accuracy, and LOD were determined from 25 standards/QC analyses with precision as the coefficient of variation (%CV) and accuracy as the percent error (%error). Acceptable criteria described previously [29] place accuracy at ≤20 % error and precision at ≤20 %CV except at the upper (≤15 %) and lower limit of quantitation (<25 %). Linearity was acceptable with a slope ≥0.8 and ≤1.2 and an *r*-squared ≥0.85. Quantifiable ranges were determined using these criteria for both 2- and 18-h reactions. Limits of detection (LOD) were determined for the method including all steps (sample preparation, enzymatic reaction, and MALDI-TOF analysis) by plotting standard deviations (SD) versus concentration for the plasma blank and three low standards, one above, one near, and one below the estimated LOD for 30 runs. The *y*-intercept and slope from the linear regression, the SD, and mean of the blank (SD_b_ and mean_b_) were used to calculate the LOD concentration as follows [30]:

$$ {\mathrm{Conc}}_{\mathrm{LOD}}=\left({\mathrm{mean}}_{\mathrm{b}}+1.645\left({s}_{\mathrm{b}}+\mathrm{i}\mathrm{n}\mathrm{t}\right)\right)/\left(1-1.645\left(\mathrm{slope}\right)\right) $$

where b is blank, *s* is standard deviation, and int is *y*-intercept.

Specificity was assessed from 100 individual NNA plasma samples and 41 archived human serum samples that included elderly Argentinian’s, children from San Diego without routine immunizations, and other infections (*Staphylococcus aureus*, *Legionella pneumophila*, *Chlamydophila pneumoniae*). Sensitivity was assessed for samples from 24 rhesus macaques (19 plasma collected, 5 serum collected) with inhalation anthrax, and in samples from three inhalation anthrax cases (CDC IRB Protocol no. 5343.0, Use of Residual Human Specimens for Laboratory Diagnostic Research) [26, 27, 31]. Samples were analyzed with two or more replicates at one or more dilutions for both 2- and 18-h reactions. Lower-level samples requiring higher sample volumes were analyzed singly due to limited sample volumes. For these, means were from both 2- and 18-h results.

Accuracy was assessed for plasma samples with 7.5, 0.75, and 0.075 ng/mL LTx co-spiked with PA83, LF, and EF at 50 ng/mL each, analyzed in triplicate. For matrix accuracy, serum and plasma from five individual donors each were spiked with LTx at 2.5 ng/mL and analyzed in duplicate. Matrix-dependent activation of PA83 to PA63 and formation of LTx was assessed using ten-donor pooled serum and plasma spiked with either 5 ng/mL PA83 or PA63 and then 2.5 ng/mL LF, incubated for 1 h then analyzed for LTx.

For utility with anthrax therapeutics, AIG interference was assessed by spiking AIG at an equivalent of 200 μg/mL anti-PA IgG into all 15 standards, plasma blank, and QC samples over the entire range from 24.3 to 0.0024 ng/mL. These were prepared, spiked, and analyzed, on two separate days alongside control (no AIG) standards/blank/QCs spiked with PBS instead of AIG. A clinical anthrax sample with previously quantified LTx of 88 ng/mL was analyzed in triplicate without and with AIG spiked at 200 μg/mL anti-PA IgG. After spiking, samples were mixed for 1 h then analyzed for LTx. Interferences from antimicrobial treatment were assessed by analyzing LTx before and after treatment for animal and clinical inhalation anthrax described above.

### Data reporting and statistical analyses

Analyses and formulas were described above where appropriate. Standard calculations, means, standard deviations, and linear regressions were performed in excel. Custom visual basic programs calculated values for LTx from the 5PL regressions. LTx levels were reported in nanograms per milliliter. Grading for culture results were described previously [25].

## Results

### Method overview

As described in the “Introduction,” the method has three primary steps, magnetic immunoprecipitation, substrate cleavage reaction, and MALDI-TOF MS quantification. The following results include characterization of LTx complex formation from PA63 and LF for standards material, characterization of the ability of the magnetic-bead-coated antibodies to selectively purify LTx, LTx reaction with substrate, and MALDI-TOF MS quantification of LTx reaction products.

### LTx standard preparation and characterization

**Fig. 2:**
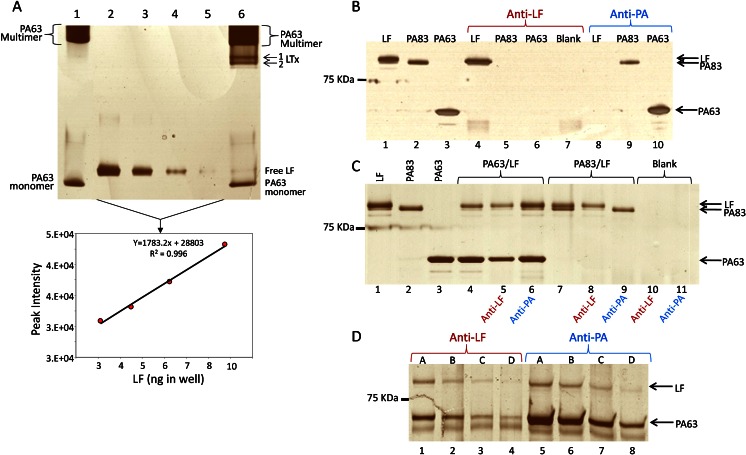
LTx and antibody characterization. LTx for standard material was analyzed by non-denaturing gel electrophoresis. The gel was scanned, LF band intensities were quantified, and the amount of free LF was estimated from the linear curve (**a**). Specificity of anti-LF and anti-PA for LF, PA83, and PA63 alone (**b**), PA63/LF (LTx) and PA83/LF (**c**), and LTx standards material; *A* 100 ng LTx, *B* 50 ng, *C* 25 ng, and *D* 12.5 ng in plasma (**d**) was assessed by magnetic immunoprecipitation and SDS-PAGE

Since the measurement of LTx relies on detection of LF activity in LTx complex, the aim was to create standards with a minimum of free LF. Activated PA63 exists as a heptamer/octamer that binds up to four LF molecules forming the LTx complex, with optimal molecular ratios represented by a weight/weight ratio of 1.5:1 PA63/LF [12, 13, 32]. However, optimum binding of LF in these standards materials required 50:1 PA63/LF as visualized by non-denaturing gel electrophoresis and higher PA63 levels did not bind more LF (not shown). PA63 alone with multimer and monomer (Fig. 2a, lane 1) was compared with the LTx mixture (lane 6) which shows two additional primary bands that migrate lower than the non-complexed multimer. These bands represent the LTx complex. A significant amount of PA63 multimer and monomer remains uncomplexed. The free LF band in lane 6 was visible, and its gel-band intensity was quantified against a linear regression derived from the LF-only lanes 2–5. The amount of free LF in the standards was quantified at 4.56 ng, which was 3 % of the total 150 ng LF loaded (Fig. 2a). LTx standard concentrations were corrected by subtracting the 3 % free LF.

### Magnetic antibody bead specificity

Antibody specificities demonstrated by magnetic immunoprecipitation of individual toxin components showed that anti-LF purified LF but not PA83 or PA63 and anti-PA purified PA83 and PA63 but not LF (Fig. 2b). Specificity for pre-formed LTx complex was observed for both anti-PA and anti-LF since both PA63 and LF bands were extracted and present (Fig. 2c). With PA83 + LF, complex was not formed and anti-LF extracted LF but not PA83 and anti-PA extracted PA83 but not LF. In all, the data support the individual specificities of anti-PA and anti-LF mAbs for LF and PA and the ability of both to purify LTx complex.

Since LTx standards were prepared with 50 times more PA63 than LF, it was determined whether the excess PA63 interfered with purification of LTx. Immunoprecipitation of 100 to 12.5 ng LTx standard material from plasma showed that the amount of LF in complex extracted by anti-PA and anti-LF MBs were visually similar (Fig. 2d). Additionally, the PA63 bands purified by anti-PA were more intense (lanes 5–8). Combined, this indicated that anti-PA levels on the beads were sufficient to capture LTx and excess non-complexed PA63. Thus, PA did not saturate the beads at these levels. Furthermore, much less material is used for LTx calibrators. The highest standard is at 24 ng/mL with only 50 μL used for analysis. In summary, anti-PA was suitable for purification of LTx and the excess PA63 did not interfere with its purification.

### Catalytic activity and quantification of LTx

**Fig. 3:**
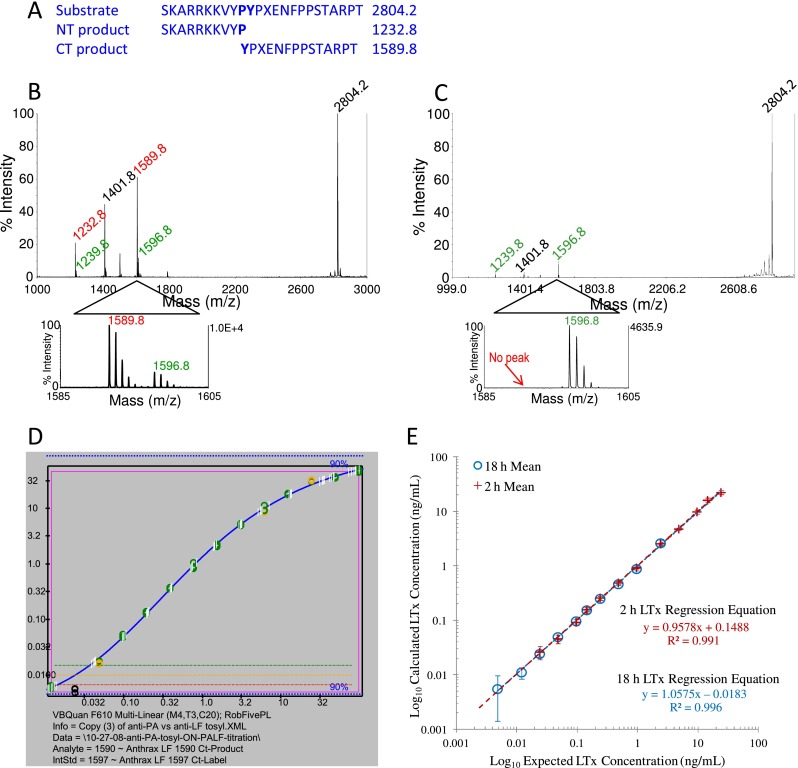
ID-MALDI-TOF MS of LTx catalytic activity and quantification. The LTx substrate peptide sequence shown in standard amino acid codes except for non-standard amino acid norleucine depicted as an *X*. Magnetic-affinity-purified LTx hydrolyzes the LF peptide substrate between the p1 proline and p1' tyrosine, yielding two smaller peptides (**a**). The cleavage reaction is mixed with ISTD peptides (+7 *m/z*) and analyzed by ID-MALDI-TOF MS. Spectra show a positive reaction with LTx in plasma (**b**) and negative reaction without LTx in plasma (**c**). Output for the 5PL regression generated by custom visual basic software for an 18-h reaction. The *orange line* indicates the estimated LOD (three times the plasma blank area ratio) for each analysis (**c**). Linearity of LTx quantification for 25 independent analyses of standards levels showing means and SD (*error bars*) for 2- and 18-h reactions (**e**)

LTx activity is quantified by cleavage of a peptide substrate yielding two products (Fig. 3a). MALDI-TOF MS of a representative positive reaction show the substrate at 2804.2 *m*/*z*, two products at 1232.8 and 1589.8 *m*/*z* and ISTD peptide peaks at 1239.8 and 1596.8 *m*/*z*, respectively (Fig. 3b). Narrowing the *x*-axis shows the isotopic detail of the CT-product and ISTD. The CT isotopic area divided by the CT-ISTD area yields the area ratio that is directly proportional to the amount of LTx present. For a negative reaction processed from the plasma blank only the substrate and ISTD peaks are present (Fig. 3c). Area ratios were plotted versus concentration and both axes were log_10_ transformed generating a sigmoidal curve. Quantitative accuracy was optimal using 5PL regression with robust weighting. Custom visual basic programs integrated MS peaks and applied the 5PL fits to quantify standards and samples (Fig. 3d).

#### LTx method validation

Performance criteria were determined with 25 independent analyses of LTx standards/QC sets. Means, standard deviations (SD), %error, and %CV are shown for each level and reaction time (Table 1). Recommended accuracy of ≤20 %error and precision of ≤20 and ≤15 %CV for the ULOQ and ≤25 %CV for the LLOQ defined the quantitative range [29, 33]. Interday accuracy for 2-h reactions was 0.1–9.8 %error from S1 to S12 ng/mL and precision was 3.7–14 %CV from S1 to S10. S11, at 24 %CV, met the recommendation for LLOQ. Therefore, the optimal quantitative range for 2- h reactions was S1–S11, with the LLOQ at 0.049 ng/mL. For 18-h reactions, accuracy and precision, respectively, were 0.2–13 %error for S5–S14 and 4.2–12 %CV for S5–S12. S13 (0.012 ng/mL) with 25 %CV, was acceptable for the 18-h LLOQ. The detection limit calculated from 30 independent analyses of low plasma standards was 0.033 ng/mL and 0.0074 ng/mL for the 2- and 18-h reaction times, respectively. The calculated mean concentrations plotted versus expected concentration showed linear regressions with slopes near 1, 0.96 for 2-h, and 1.06 for 18-h reactions, and *r* ^2^ ≥ 0.991 for both 2- and 18-h reactions (Fig. 3e). Mean values for 2- and 18-h reactions were precisely overlaid at the universal levels. Precision from 25 independent QC analyses was 9.9 and 7.2 %, respectively, for QCH and QCM for the 2-h reaction time and 6.3 and 9.0 %, respectively for QCM and QCL for the 18-h reaction time (Table 1).

**Table 1:** Mean, SD, accuracy (% error), precision (%CV), and analytical ranges (bold font) for LTx standards, plasma blank (PBL), and quality control samples (QCs), for 2- and 18-h reactions analyzed over 25 days. Standard levels with ≤20 % error, ≤20 %CV for middle range, ≤15 % CV (ULOQ), and ≤25 % CV (LLOQ) are indicated in italics. Limits of detection (LOD) were calculated as described

LTx standard	Expected (ng/mL)	2-h reaction				18-h reactions			
		Observed (ng/mL)	SD	%error	CV (%)	Observed (ng/mL)	SD	%error	CV (%)
S1	24.3	**22.9**	1.78	*5.4*	*7.8*	14.0	4.3	42	31
S2	14.6	**16.0**	1.47	*9.8*	*9.2*	12.9	1.9	11	15
S3	9.7	**9.55**	0.56	*1.5*	*5.9*	11.6	2.6	20	23
S4	4.85	**4.81**	0.24	*0.9*	*4.9*	5.89	1.2	21	20
S5	2.43	**2.52**	0.093	*4.1*	*3.7*	**2.63**	0.31	*8.3*	*12*
S6	0.97	**0.932**	0.042	*3.9*	*4.5*	**0.890**	0.051	*8.2*	*5.7*
S7	0.485	**0.485**	0.018	*0.1*	*3.7*	**0.462**	0.019	*4.8*	*4.2*
S8	0.243	**0.254**	0.019	*4.8*	*7.4*	**0.249**	0.015	*2.7*	*6.1*
S9	0.146	**0.156**	0.016	*7.0*	*11*	**0.155**	0.010	*6.7*	*6.4*
S10	0.097	**0.093**	0.013	*4.0*	*14*	**0.097**	0.0071	*0.2*	*7.3*
S11	0.049	**0.045**	0.011	*7.2*	*24*	**0.050**	0.0050	*2.1*	*10*
2-h LOD		**0.033**	NA	NA	NA				
S12	0.024	0.024	0.0083	*0.6*	34	**0.024**	0.0029	*0.4*	*12*
S13	0.012	0.014	0.0067	*18*	47	**0.011**	0.0027	*13*	*25*
18-h LOD						**0.0075**	NA	NA	NA
S14	0.0049	0.009	0.0078	83	88	0.0054	0.0039	*12*	72
S15	0.0024	0.007	0.0074	185	107	0.0035	0.0014	45	39
PBL	0	0.0019	0.0041	NA	213	0.00037	0.0010	NA	278
QCH	5.82	5.29	0.52	*9.1*	*9.9*	NA	NA	NA	NA
QCM	0.582	0.547	0.039	*6.0*	*7.2*	0.528	0.033	*9.3*	*6.3*
QCL	0.0582	NA	NA	NA	NA	0.055	0.005	*5.3*	*9.0*

Extraction recoveries could not be properly assessed because activity of LF was higher when bound by PA63 and anti-PA-MB. Area ratios obtained from reactions were much lower for 0.5 ng LF alone (1.4 ± 0.02), compared with 0.5 ng LF in complex as LTx (20 ± 1) and LTx extracted by anti-PA (22 ± 0.3). This implied recoveries of >100 %.

Specificity was 100 %. There were no false positives for 100 plasma samples from North American donors and 41 serum samples from other infections. Sensitivity was 100 % with LTx measured in all infected subjects, three documented inhalation anthrax cases, and 24 rhesus macaques [26, 27, 31, 34].

There was no observable interference from other anthrax toxins and accuracy was good with low error of 0.8–12.7 % for samples covering three orders of magnitude (Table 2). This included 50 ng PA83 and EF toxins, 667-fold excess at the lowest LTx level. Inclusion of 50 ng/mL free LF contributed to higher LTx as expected since it was able to bind the excess free PA63 in the LTx sample.

**Table 2:** Accuracy (%error) of LTx quantitation in the presence of other anthrax toxins, PA83, LF, and EF

	LTx (ng/mL)	PA83 (ng/mL)	LF (ng/mL)	EF (ng/mL)	Mean ± SD (2 h)	Mean ± SD (18 h)	%error (2 h)	%error (18 h)
LTx-1	7.5	–	–	–	8.2 ± 0.31		8.5	
LTx-2	7.5	50	–	–	8.4 ± 0.17		11.5	
LTx-3	7.5	–	50	–	22 ± 0.19		NA	
LTx-4	7.5	–	–	50	7.7 ± 0.15		3.3	
LTx-5	0.75	–	–	–	0.80 ± 0.022	0.71 ± 0.023	6.1	5.9
LTx-6	0.75	50	–	–	0.84 ± 0.011	0.74 ± 0.011	12.3	0.8
LTx-7	0.75	–	50	–	8.5 ± 0.12	8.2 ± 0.38	NA	NA
LTx-8	0.75	–	–	50	0.76 ± 0.03	0.66 ± 0.021	1.2	11.8
LTx-9	0.075	–	–	–	<LOD	0.083 ± 0.0035		9.9
LTx-10	0.075	50	–	–	<LOD	0.085 ± 0.0012		12.7
LTx-11	0.075	–	50	–	0.98 ± 0.018	0.86 ± 0.032		NA
LTx-12	0.075	–	–	50	<LOD	0.076 ± 0.0022		1.8

Matrix-dependent accuracy was assessed because serum and plasma samples may have different components released during processing that influence LTx activity. LTx levels measured in the serum samples (2.6 ± 0.28 ng/mL) were similar to plasma (2.4 ± 0.17 ng/mL) for a 2-h reaction but were higher in serum (3.1 ± 0.42 ng/mL) than plasma (2.6 ± 0.31 ng/mL) after 18 h. The mean %error in calculated value from the expected 2.5 ng/mL for 2 and 18 h combined was lower for plasma samples at 7.7 ± 6.8 % compared with serum at 17 ± 14 % suggesting that factors present in serum may reduce accuracy and produce more variability in LTx activity.

Serum and plasma matrices were also evaluated for artificial cleavage of PA83 to PA63 by serum clotting cascade proteases which would form LTx in the presence of LF. No LTx was detected in serum or plasma spiked with PA83 and LF at 5 and 2.5 ng/mL, respectively. In contrast, PA63 and LF spiked together resulted in significant formation of complex in both serum (1.8 ± 0.16 ng/mL) and plasma (2.0 ± 0.074 ng/mL), with plasma LTx measured higher than serum. This demonstrated that no measureable conversion of PA83 to PA63 occurred in serum or plasma under these conditions.

The interference of AIG (Cangene Corp, Canada) on LTx quantification was assessed by spiking standards, QCs, plasma blank, and an infection sample with therapeutic levels of AIG (200 μg/mL anti-PA IgG). LTx measured was lower with AIG compared with samples without AIG (Table 3). Overall, accuracy was high with only 9.4 ± 8.0 % error without AIG and was lower with AIG at 40 ± 20 % error. However, accuracy was better for the higher AIG-spiked standards, 11–24 % error (S2–S4) and was reduced in lower standards and all QCs, ranging from 34 to 70 % error (S5–S12, QCH, M, L). LTx with AIG was detected as low as the S12 standard (0.024 ng/mL), though it was quantified lower at 0.014 ± 0.007 ng/mL. LTx in a clinical sample was similar at 93 ± 4.7 ng/mL with AIG compared with that without AIG (88 ± 2.6 ng/mL), indicating no interference by AIG in this sample (Table 3).

**Table 3:** Effect of AIG (200 μg/mL) on LTx quantification

Level	Expected (ng/mL)	No AIG			AIG		
		LTx (ng/mL)	SD	%error	LTx (ng/mL)	SD	%error
S1	24.3	22	5.20	9.0	17	0.79	32
S2	14.6	13	2.84	10	13	2.3	11
S3	9.7	11	1.34	16	8.5	2.8	12
S4	4.85	4.1	1.68	17	3.7	1.8	24
S5	2.43	2.6	0.108	8.1	1.6	0.68	35
S6	0.97	0.87	0.008	10	0.41	0.033	57
S7	0.485	0.48	0.014	1.9	0.20	0.014	58
S8	0.243	0.26	0.020	5.3	0.14	0.042	41
S9	0.146	0.16	0.030	11	0.096	0.029	34
S10	0.097	0.099	0.0035	2.5	0.042	0.012	57
S11	0.049	0.049	0.0060	0.03	0.016	0.0080	68
S12	0.024	0.018	0.0008	24	0.014	0.0071	42
S13	0.012	0.013	0.0017	9.0	<LOD	NA	NA
S14	0.0049	<LOD	NA	NA	<LOD	NA	NA
S15	0.0024	<LOD	NA	NA	<LOD	NA	NA
Plasma BL	0	<LOD	NA	NA	<LOD	NA	NA
QCH	5.82	7.4	1.2	28	3.1	0.029	47
QCM	0.582	0.54	0.021	7.4	0.29	0.051	51
QCL	0.0582	0.06	0.001	0.21	0.02	0.010	70
Sample	88	88	2.6	0.23	93	4.7	5.7
Mean ± SD				9.4 ± 8.0			40 ± 20

Mean, SD, and accuracy (%error) for standards, QCs, and a clinical anthrax sample are shown

Interference of LTx detection by antimicrobial treatment was assessed in six rhesus macaques and three clinical cases [26, 27, 31, 34]. Pre- and post-antimicrobial treatment LTx levels were determined for all subjects except one for which a pre-treatment sample was not available (Table 4). Pre-treatment LTx levels were 2.3–280 ng/mL. LTx was detected post-treatment in all subjects. Latest post-treatment LTx was low in the survivors (2–8 days post-treatment), at 0.012–0.096 ng/mL, and was higher in two non-surviving rhesus macaques, 8.6 and 8.7 ng/mL. In animal R050003, the higher post-treatment LTx appeared to be due to a short treatment interval (36 h) and in animal 04208 to higher pre-treatment LTx (280 ng/mL).

**Table 4:** Impact of antimicrobial agents on LTx detection during inhalation anthrax in rhesus macaques treated with Ciprofloxacin (Cipro) commencing at the time point indicated 48 or 72 h post-challenge (PC) and in human clinical cases treated with antimicrobial agents following hospitalization

Subject	Status	Antimicrobials	Pre-Abx LTx (ng/mL)	Sample time pre-Abx	Last post-Abx LTx (ng/mL)	Sample time post-Abx
RM (R050003)	NS (72 h)	Cipro 48 h PC	14	36 h PC	8.6	36 h (LA)
RM (04208)	NS (6 days)	Cipro 48 h PC	280	48 h PC	8.7	72 h (LA)
RM (03E062	S	Cipro 48 h PC	7.6	48 h PC	0.095	48 h
RM (04E067)	S	Cipro 48 h PC	12	48 h PC	0.096	72 h
RM (0412)	S	Cipro 72 h PC	8.3	72 h PC	0.072	72 h
RM (04E083)	S	Cipro 72 h PC	2.3	72 h PC	0.12	72 h
Human (2006)	S	At admission	2.6	3DPO	0.012	8 days
Human (2008)	NS	At admission	NA	NA	0.018	6 days (FA)
Human (2011)	S	At admission	2.3	2DPO	0.023	68 h

The LTx results are given for samples obtained prior to antimicrocrobial treatment (Pre-Abx) and the last available sample positive (post-Abx)

*NA* not available, *NS* non-survival, *S* survived, *LA* latest available post-antibiotic sample, *FA* first available, *Abx* antimicrobials/antibiotics, *DPO* days post-symptom onset

#### LTx detection in rhesus macaques during inhalation anthrax

Two female rhesus macaques, 16N and 89N, were exposed by aerosol inhalation to 248 and 264 LD_50_ *B. anthracis* Ames spores, respectively. Both animals survived 5 days post-exposure. Both were positive for culture, *pagA* PCR, and LTx by 36 h and remained positive (Table 5). Earliest LTx levels were low, 0.24 and 0.10 ng/mL for 16N and 89N, respectively. Animal 16N increased almost 100-fold from 36 to 72 h to 21 ng/mL, declined to 5.4 ng/mL at 96 h, then increased >2000-fold at 120 h to 12,100 ng/mL. Animal 89N increased 10-fold from 36 to 48 h to 1.02 ng/mL, remained stable at 72 h, increased 10-fold at 96 h (11.5 ng/mL), then 100-fold at 120 h (1150 ng/mL).

**Table 5:** Comparison of LTx quantification with other anthrax diagnostic biomarkers, bacteremia (bact), *pagA* PCR, and total LF, over the course of inhalation anthrax in two rhesus macaques

Time PC	16N			89N		
	Bact/PCR	LTx	Total LF	Bact/PCR	LTx	Total LF
−30 days	–	<LOD	<LOD	–	<LOD	<LOD
12 h	–	NT	<LOD	–	NT	<LOD
18 h	–	NT	0.026	–	NT	<LOD
24 h	–	<LOD	0.13 ± 0.016	–	NT	0.049 ± 0.012
30 h	–	<LOD	0.69 ± 0.044	–	<LOD	0.32 ± 0.061
36 h	±/+	0.24 ± 0.041	1.8 ± 0.16	+/+	0.10 ± 0.012	2.2 ± 0.51
48 h	+/+	0.76 ± 0.074	13 ± 1.0	+/+	1.02 ± 0.039	30 ± 1.5
72 h	+/+	21 ± 0.54	53 ± 3.7	+/+	1.25 ± 0.079	20 ± 0.64
96 h	NA	5.4 ± 1.4	22 ± 0.35	NA	12 ± 1.5	72 ± 2.7
120 h	+/+	12,100 ± 1270	12,200 ± 670	+/+	1150 ± 170	1930 ± 44

Bact is positive (+) for ≥6 colonies in the primary streak, low positive (±) for one to five colonies, negative (−) for no colonies. Qualitative *pagA* PCR is positive (+) or negative (−). LOD = 0.0075 ng/mL for LTx and 0.005 ng/mL for total LF. All samples with available volumes were analyzed in duplicate at minimum and at one or more dilutions

*PC* post-challenge, *NA* not analyzed

Comparison of LTx with total LF (free LF + LTx) showed that LF was first detected in animal 16N and 89N at 18 and 24 h post-exposure, respectively, 18 and 12 h prior to LTx, culture, and PCR (Table 5). At 36 h, LF was 7.5–22 times higher than LTx, demonstrating a majority of free LF in the earliest stages of infection. However, at the terminal stage, LTx increased and was 100 % of the total LF for 16N and 60 % for 89N. In summary, LTx was detected early and increased to become the dominant form of LF in late infection.

## Discussion

Here, we report development and validation of a method that quantifies LTx complex. Combining the strategy that provided sensitive LF detection with selective PA purification allowed sensitive measurements for LTx. It is more sensitive, 0.033 ng/mL for 2 h and 0.0075 ng/mL for the 18-h reaction, and more accurate than other methods for PA [2–9]. The two reaction times provide quantitation over a large dynamic range, from 24 to 0.0012 ng/mL, which is extended even higher with sample dilutions, as seen with the highest infection sample at 12,100 ng/mL. With a 2-h reaction, the method achieves a relatively fast 4-h total time to detection and is still extremely sensitive compared with other methods. The longer incubation time improves the detection limit 4.7-fold and assists in confirming low-level positives, ruling out negatives and confirmation of low-level quantification.

Accuracy within the defined quantifiable ranges was high (0.1–13 % error) under all conditions in plasma, including those with other toxins expected to be present during infection. In a less optimal matrix such as serum, accuracy was acceptable (17 ± 14 % error) but had greater variability compared with plasma (7.7 ± 6.8 %). This may be due to serum collection that can include varying degrees of neutrophil lysis and release of defensins which are reported to decrease LF activity [35]. Serum preparation may also initiate the clotting cascade that releases proteases that could artificially cleave and activate PA83 to PA63, artificially increasing LTx measured. This was not observed when PA83 was spiked along with LF in either serum or plasma. There was no consistency in whether LTx measured in serum was increased or decreased. When spiked in various donor samples, LTx was higher in serum than plasma. When spiked with PA63 and LF separately, LTx was lower in serum than plasma. For LTx quantification, either matrix can be used but plasma is preferred.

Three steps, antibody purification, enzymatic activity, and mass spectrometric detection of specific cleavage products, combine to minimize cross reactivity. Taken together, it provided specificity of 100 %. Sensitivity was also 100 % with no false negatives for 3 humans and 24 rhesus macaque inhalation infections. Though the human inhalation anthrax case numbers for which samples were available was low, the sensitivity was supported by the positive results in 24 inhalation-infected rhesus macaques and was further supported by positive results following antibiotic treatment in all nine available treated subjects and as long as 8 days post-antibiotics in one case [26]. This is also important in terms of diagnostic potential, since antibiotic use renders methods that depend on the organism such as culture and PCR, negative, usually within 24 h following treatment initiation.

This method may be useful for evaluation of the efficacy of PA-targeted therapeutics such as AIGIV. There was higher error of 40 ± 20 % from AIG at LTx levels below 1 ng/mL, which was similar to that seen previously for a TRF PA assay with a 50 % reduction in signal for a 5.4-fold ratio of AIG to PA [5]. However, LTx was still detectable at low levels, 0.024 ng/mL, even with extreme excesses of 200 μg/mL anti-PA IgG. Thus, any negative impact on detection was small in context of the detection limit. There was no interference in an AIG-spiked clinical sample. Though it reduces the ability to accurately measure LTx at lower levels, in vitro, it is expected that during treatment, AIG binding PA63 would actually reduce circulating levels in vivo. The in vitro interference could be a measure of in vivo LTx clearance. The utility of this method for AIG will be validated further.

Previous reports of freely circulating LTx complex in terminal samples in rabbits, guinea pigs, and monkeys [18, 20, 36], were confirmed in this study in rhesus macaques. The sensitivity of this method allowed the first detection of catalytically active LTx early and throughout infection. That LTx increases to high levels in late infection, suggests its importance in the process of infection and rapid death. In all, these results demonstrated the importance of LTx as a diagnostic and therapeutic target. The method described should provide a sensitive, fast, accurate tool for anthrax toxin detection and evaluation of PA-directed therapeutics.

## Abbreviations

5PL: Five-parameter logistic
AIGIV: Anthrax immune globulin intravenous
ID: Isotope dilution
ISTD: Internal standard
LF: Lethal factor
LOD: Limit of detection
LTx: Lethal toxin
MALDI-TOF MS: Matrix-assisted laser desorption ionization time-of-flight mass spectrometry
MB: Magnetic beads
NNA: Normal North American
PA: Protective antigen
PCR: Polymerase chain reaction
SD: Standard deviation
